# Dextroamphetamine Treatment in Children With Hypothalamic Obesity

**DOI:** 10.3389/fendo.2022.845937

**Published:** 2022-03-09

**Authors:** Jiska van Schaik, Mila S. Welling, Corjan J. de Groot, Judith P. van Eck, Alicia Juriaans, Marcella Burghard, Sebastianus B. J. Oude Ophuis, Boudewijn Bakker, Wim J. E. Tissing, Antoinette Y. N. Schouten-van Meeteren, Erica L. T. van den Akker, Hanneke M. van Santen

**Affiliations:** ^1^ Division of Pediatric Endocrinology, Wilhelmina Children’s Hospital, University Medical Center Utrecht, Utrecht, Netherlands; ^2^ Department of Pediatric Oncology, Princess Máxima Center for Pediatric Oncology, Utrecht, Netherlands; ^3^ Obesity Centre Centrum Gezond Gewicht (CGG), Erasmus Medical Center (MC) Sophia Children’s Hospital, University Medical Center Rotterdam, Rotterdam, Netherlands; ^4^ Division of Pediatric Endocrinology, Erasmus Medical Center (MC) Sophia Children’s Hospital, University Medical Center Rotterdam, Rotterdam, Netherlands; ^5^ Department of Exercise Physiology, Child Development & Exercise Center, Wilhelmina Children’s Hospital, University Medical Center Utrecht, Utrecht, Netherlands; ^6^ Department of Pediatric Psychiatry, Wilhelmina Children’s Hospital, University Medical Center Utrecht, Utrecht, Netherlands; ^7^ Department of Pediatric Oncology/ Hematology, University of Groningen, University Medical Center Groningen, Groningen, Netherlands

**Keywords:** hypothalamic obesity, craniopharyngioma, genetic obesity, dextroamphetamine, resting energy expenditure

## Abstract

**Introduction:**

Hypothalamic obesity (HO) in children has severe health consequences. Lifestyle interventions are mostly insufficient and currently no drug treatment is approved for children with HO. Amphetamines are known for their stimulant side-effect on resting energy expenditure (REE) and suppressing of appetite. Earlier case series have shown positive effects of amphetamines on weight in children with acquired HO. We present our experiences with dextroamphetamine treatment in the, up to now, largest cohort of children with HO.

**Methods:**

A retrospective cohort evaluation was performed of children with HO treated with dextroamphetamine at two academic endocrine pediatric clinics. Off-label use of dextroamphetamine was initiated in patients with progressive, therapy-resistant acquired or congenital HO. Anthropometrics, REE, self-reported (hyperphagic) behavior and energy level, and side effects were assessed at start and during treatment.

**Results:**

Nineteen patients with a mean age of 12.3 ± 4.0 years had been treated with dextroamphetamine. In two patients, ΔBMI SDS could not be evaluated due to short treatment duration or the simultaneous start of extensive lifestyle treatment. Mean treatment duration of the 17 evaluated patients was 23.7 ± 12.7 months. Fourteen patients (*n* = 10 with acquired HO, *n* = 4 with congenital HO) responded by BMI decline or BMI stabilization (mean ΔBMI SDS of -0.6 ± 0.8, after a mean period of 22.4 ± 10.5 months). In three patients, BMI SDS increased (mean ΔBMI SDS of +0.5 ± 0.1, after a mean period of 29.7 ± 22.6 months). In 11 responders, measured REE divided by predicted REE increased with +8.9%. Thirteen patients (68.4%) reported decreased hyperphagia, improvement of energy level and/or behavior during treatment. Two patients developed hypertension during treatment, which resulted in dosage adjustment or discontinuation of treatment. Twelve children continued treatment at last moment of follow-up.

**Conclusion:**

In addition to supportive lifestyle interventions, dextroamphetamine treatment may improve BMI in children with HO. Furthermore, dextroamphetamines have the potential to decrease hyperphagia and improve resting energy expenditure, behavior, and energy level. In patients with acquired HO, these effects seem to be more pronounced when compared to patients with congenital HO. Future studies are needed to support these results.

## Introduction

Hypothalamic dysfunction (HD) in children may have devastating consequences. HD can lead to an impaired satiety regulation (i.e. hyperphagia), decreased energy expenditure, and/or a distorted sympathetic activity (1–6). All in all, these processes induce progressive weight gain resulting in severe hypothalamic obesity (HO) and a higher risk of cardiovascular problems at a later age (7). Moreover, HD results in neurocognitive and physiological morbidity, reducing quality of life in these children (8).

The hypothalamus regulates both food intake and energy expenditure. Maintenance of an optimal balance in body weight is the result of a complex interaction of, amongst others, the different hypothalamic nuclei. This complex network has been widely investigated in animal, including rodent (9), and human studies (10). The ventromedial hypothalamus (VMH) and arcuate nucleus (AN) regulate hunger, satiety, and energy balance (11). Insulin, ghrelin, Glucagon-like Peptide-1 (GLP-1), and leptin are key hormones in the regulation of energy balance and signaling to the VMH and AN (12, 13). In addition to these pathways, other systems, such as the hedonic system, are also involved in regulating food intake and influenced by the hypothalamus (6). In rare cases, the hypothalamus malfunctions during childhood (14). This can either be due to a congenital cause, such as gene defects affecting the hypothalamic leptin-melanocortin pathway, or an acquired cause, due to (treatment of) a suprasellar tumor, trauma, or a cerebral infection (1, 4, 15). This hypothalamic malfunctioning can lead to increased feeding and/or decreased energy expenditure resulting in invariably severe obesity, as shown in rodent studies (16, 17), as well as human studies (18).

Currently, no effective treatment is available and approved for children with HO. Lifestyle interventions, applied as diet and/or exercise interventions, are often insufficient, because of the inexorable hyperphagia that results from HD (1, 15, 19). Consequently, there is a need for additional therapeutic options, such as pharmacotherapy. One of the viable pharmaceuticals for such an intervention could be (dextro)amphetamines. In the past, these psychostimulants have been regularly prescribed for obesity treatment. However, some decades later, the use of amphetamines for obesity treatment was restricted, due to its addictive potential, side effects, and lack of long-term effect (20). Currently, (dextro)amphetamines are often used in treating attention-deficit-hyperactivity disorder, where decreased weight due to reduced appetite is often an unintended side-effect (21).

Amphetamines influence both the hypothalamic and hedonic system by increasing the concentration of two important energy balance related neurotransmitters, i.e. dopamine and serotonin, affecting food intake as well as resting energy expenditure (REE) (22–24). Moreover, they are sympathomimetic drugs which directly stimulate cocaine-amphetamine regulated transcript (CART) neurons of the leptin-melanocortin pathway in the arcuate nucleus and lateral hypothalamic area, leading to an increased α-MSH production which attenuates hyperphagia and improves satiety (11, 12, 25). By specifically targeting the hyperphagia and reduced REE, amphetamines could have the potential to significantly ameliorate the distressing effects of hyperphagia and low energy expenditure leading to progressive weight gain in patients with HD (26–28).

Studies on the effect of dextroamphetamines in HO are limited and mostly done in children with acquired HO (26–28). Data on the effect of this treatment in children with congenital HO are, however, lacking. Moreover, the effects of dextroamphetamines on energy expenditure and body composition have not been described previously in children with HO. During the past years, we have offered off-label dextroamphetamine treatment to patients with severe therapy-resistant obesity due to acquired or congenital HO. Here, we present the effects of dextroamphetamine treatment in children with hypothalamic obesity who were treated in two academic endocrine pediatric clinics.

## Methods

### Study Population and Treatment

All children and young adolescents diagnosed with HO, either due to congenital or acquired brain injury, and treated with dextroamphetamine at two academic endocrine pediatric clinics between 2014 and 2021 are retrospectively described. In patients, off-label dextroamphetamine treatment was initiated when hyperphagia and severe therapy-resistant hypothalamic obesity were present. Patients and their parents were informed comprehensively about the off-label use and (potential) side effects of dextroamphetamine. Treatment was given in the context of outpatient clinical care. Initial dextroamphetamine dosing was daily 5 mg. This was increased weekly with 5 mg/day (maximum of 0.5 mg/kg/day and until maximum dose of 40 mg/day) depending on the effect on weight, hyperphagia, and side effects. Patients were instructed to continue their personalized dietary and/or physical activity plan. During the visits at the outpatient clinic, the effect of dextroamphetamines was evaluated and patients continued treatment when the positive effects outweighed the negative (side) effects. If not, treatment was discontinued.

### Data Collection

All data were retrospectively collected. Patients visited the outpatient clinic at least four times per year (at three months, six months, nine months, and one year after start therapy). During each visit, weight and height were measured. Weight was rounded up or down to the nearest 100 grams and height to the nearest millimeter. Self-reported parameters were evaluated: therapy adherence of dextroamphetamine, and endocrine substitution, hyperphagia or food seeking behavior, other behavioral issues (e.g. concentration, number of rage attacks), energy level during the day, sleep pattern (e.g. falling asleep, sleep efficiency, daytime somnolence), and physical activity during the day. REE and body composition were assessed prior and during treatment. Of the children with acquired HO, data on endocrine parameters (free thyroxine (FT4), insulin-growth factor (IGF-1) SDS) and hydrocortisone orally (mg/m^2^/day) was collected from prior (the year before treatment) and during treatment. Since data was collected retrospectively and in the context of clinical care, follow-up intervals and availability of clinical data varied.

### Resting Energy Expenditure and Body Composition

REE was measured after an 8-h overnight fast and after a 30 minute resting period, using indirect calorimetry. This was done with either the Cosmed Quark RM (Cosmed, Italy) or Geratherm Ergostik (Geratherm Respiratory GmbH, Germany). For each patient, the predicted REE (pREE) was calculated using the Schofield equation based on age, sex, weight, and height (29). The percentage of the quotient of measured REE (mREE) divided by predicted REE was calculated (mREE/pREE * 100%). Body composition, i.e. fat mass percentage (FM%), was measured either using dual energy X-ray absorptiometry (DEXA) scans, air displacement plethysmography (BOD POD, Cosmed, Italy) or with a Body Impedance Analysis (BIA, using the Bodystat 1500 or Tanita). For both REE and body composition measurements, preferably, the same technique was used at start and during treatment in each patient.

### Adverse Effects

Presence of adverse effects during treatment was assessed at every visit by history taking and physical examination, including measurement of blood pressure, with special interest for hypertension (defined as systolic or diastolic blood pressure >95^th^ percentile, adjusted for age, sex, and height), cardiovascular complaints (palpitations, tachycardia), behavioral problems (aggressiveness), or neurological complaints (headache, restlessness, insomnia) (30).

### Statistical Analysis

For children and adolescents, BMI (calculated by weight in kilograms divided by height in meters squared) is age- and sex-specific, therefore BMI standard deviation scores (BMI SDS) were calculated using Dutch references for sex and age (31, 32). Primary outcome was change in BMI SDS over time (ΔBMI SDS). BMI SDS trajectories one year before and after treatment (only for the linear mixed model analysis) were compared to the period of receiving treatment using a cross-sectional analysis (at group levels and in subgroups) and linear mixed model analysis (at group level). The linear mixed model was not adjusted for other variables. Being treated was defined as receiving dextroamphetamine for at least one month. Not being treated was defined as not receiving dextroamphetamine for at least one month. Subgroups were made based on their response to treatment during the first year of treatment.

Responders were classified as either belonging to the BMI decline subgroup, defined as having a decrease of ≥-0.25 in BMI SDS during the first year of treatment, or belonging to the BMI stabilization subgroup, defined as having a change of <-0.25 to 0.10 in BMI SDS during the first year of treatment. Non responders were defined as having an increase of ≥+0.10 BMI SDS during the first year of treatment. Mean or medians of BMI SDS, hormonal supplementation, and serum concentrations of endocrine parameters such as FT4 and IGF1 (for acquired HO patients) prior to start and during treatment, were compared using paired-T test or Wilcoxon-signed rank test depending on the distribution. Statistical analysis was performed using SPSS version 25.0 [IBM]. Data are presented as mean and standard deviation or as ranges (minimum-maximum). A p-value of < 0.05 was considered significant.

### Ethics

Because of the retrospective nature of the study, the local institutional review board decided that the Act on Medical Research Involving Human Subjects did not apply and provided a waiver. Informed consent for participation and publication of the data was obtained from all parents and/or patients.

## Results

### Baseline Characteristics

In total, 19 patients, of which 14 females, had been given dextroamphetamine treatment for hypothalamic obesity. Twelve children were diagnosed with acquired HO, five children with a molecularly proven genetic obesity, one child with a Prader-Willi-like phenotype without a molecularly proven diagnosis of genetic obesity, and one child was suspected for HO due to weight gain after anatomical brain injury caused by Chiari II malformation and hydrocephalus for which an ventriculoperitoneal shunt was placed.

Mean age at start of treatment was 12.3 ± 4.0 years and mean dextroamphetamine treatment duration was 23.7 ± 12.7 months [9.8 - 54.2]. Seven children (36.8%) had grade I obesity (comparable with BMI > 30 kg/m2 in adults), five children (26.3%) had grade II obesity (comparable with BMI > 35 kg/m2 in adults), and seven children (36.8%) had grade III obesity (comparable with BMI > 40 kg/m2 in adults). Two patients (ID 1 and 4) participated in another study in parallel which entailed additional extensive dietary coaching. One of these patients (ID 1) also started with additional exenatide for one year, 16 months after the start of dextroamphetamines, without beneficial effects on weight (33). This patient also started additional oxytocin nasal spray for 1.5 year, two years after the start of dextroamphetamine, again without any effect on weight. One patient (ID 7) already had maintenance oxytocin nasal spray and continued this treatment throughout the period of dextroamphetamine treatment. Parents had reported positive effects of oxytocin treatment on behavior (less agitated, less emotional, and less hyperphagia). At end of follow-up, twelve patients (10 patients with acquired HO and 2 patients with genetic HO) continued dextroamphetamine treatment. Baseline characteristics are depicted in **Table 1**.

**Table 1:** Patient characteristics at baseline.

Patient ID	Sex	Diagnosis	Age at diagnosis(years)	Age at start dextroamphetamines (years)	Starting/highest dose of dextroamphetamine (mg)
*Acquired hypothalamic obesity*					
1	F	Craniopharyngioma	5.7	12.8	10/15
2	F	Craniopharyngioma	3.5	7.1	2.5/10
3	M	Craniopharyngioma	4.9	15.3	5/25
4	F	Craniopharyngioma	10.7	14.8	5/15
5	M	Low grade glioma	5.0	8.3	5/22.5
6	F	Craniopharyngioma	7.3	16.1	15/30
7	M	Low grade glioma	1.2	8.3	5/5
8	M	Low grade glioma	0.6	9.1	5/15
9	F	Craniopharyngioma	6.5	10.2	5/10
10	F	Craniopharyngioma	5.5	14.3	5/40
11	F	Craniopharyngioma	9.7	11.6	10/20
12	F	Neonatal meningitis	0.3	9.6	10/35
*Congenital hypothalamic obesity*					
13	F	Pseudohyperparathyreoidism-1a	6.3	13.6	10/10
14	F	Bardet-Biedl syndrome 1	10.0	17.1	10/20
15	F	Leptin receptor deficiency	14.9	18.0	10/70
16	M	Prader Willi like^a^	n.a.	15.8	10/22.5
17	F	Temple syndrome	6.0	14.6	10/45
18	F	Leptin receptor deficiency	1.3	2.7	10/15
19	F	Chiari-II malformation, hydrocephalus	0.0	14.8	10/20

F, female; M, male; n.a., not available.

a clinical phenotype of early-onset severe obesity, autism, intellectual deficit, congenital deformations compatible with VACTERL-association, without genetic diagnosis after extensive DNA diagnostics.

Of the 12 children with acquired HO, 11/12 had hypothalamic damage due to a suprasellar tumor (*n* = 11). At diagnosis of their suprasellar tumor, three out of these 12 children (25.0%) presented with underweight, four (33.3%) with normal weight, one (8.33%) with overweight and three (25.0%) with obesity. All children with a suprasellar tumor had been treated with neurosurgery, of which five children underwent a gross total resection and six children a partial resection. Additionally, three of these children (27.2%) had been treated with radiotherapy with a cumulative dose of 54 Gy and three other children (27.2%) had been treated with chemotherapy. Nine children (81.8%) had experienced progression of their residual tumor during follow-up. However, all children were in stable disease or remission at time of starting dextroamphetamine treatment. Mean ΔBMI SDS from diagnosis of the brain tumor until start of treatment was +2.75 ± 2.83, with a mean follow-up time of 6.54 ± 2.64 years.

### Effect of Dextroamphetamines on BMI SDS on Group Level

In 17 out of 19 patients, the effects of dextroamphetamine treatment on BMI could be evaluated. One patient with genetic HO was excluded from evaluation, due to the fact that a first extensive lifestyle treatment at a rehabilitation clinic was started in parallel with dextroamphetamine treatment (ID 18). In the other patient, also with genetic HO, dextroamphetamine treatment was ended after one month due to hypertension (multiple measurements showed systolic blood pressure >95^th^ percentile) (ID 14).

**Figure 1:**
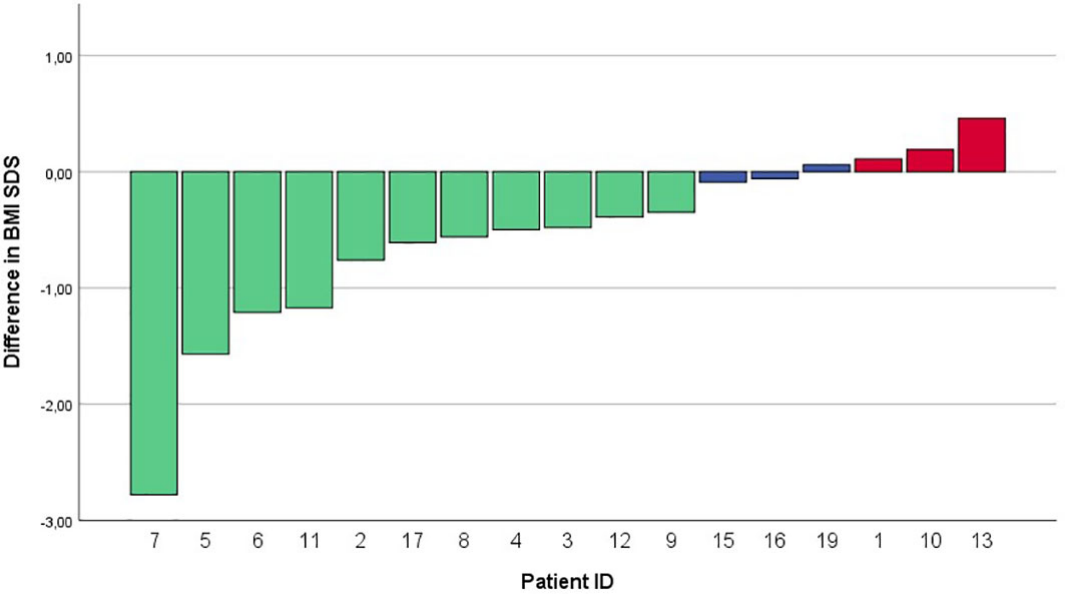
Waterfall plot of differences in BMI SDS between start of treatment and after 12 months of ongoing treatment. BMI SDS, Body Mass Index standard deviation score.

**Figure 2:**
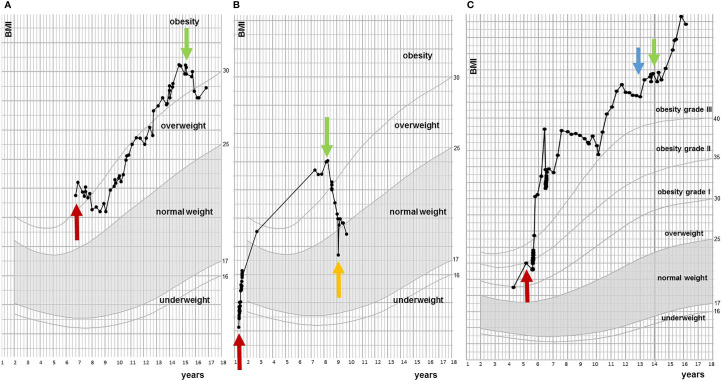
Examples of BMI trajectories during course of dextroamphetamine treatment. **(A)** Responder. Red arrow indicates first BMI measurement at pediatric center two years after diagnosis of craniopharyngioma and green arrow indicates start of dextroamphetamine. **(B)** Responder. Red arrow is moment of diagnosis (low grade glioma), green arrow indicates start of dextroamphetamine treatment and orange arrow is stop of dextroamphetamine. **(C)** Non-responder. Red arrow is moment of diagnosis (craniopharyngioma), blue arrow indicates start of exenatide treatment and green arrow is start of dextroamphetamine treatment.

Of the 17 evaluated patients, mean BMI SDS at start of treatment was 3.58 ± 0.85 [range 2.46 – 5.05], which decreased to 3.24 ± 1.07 after 6 months of treatment [range 1.39 – 5.10], and to 3.18 ± 1.44 [0.95 – 5.68] at last moment of follow-up (**Table 2**). In the year before start of treatment (mean year 1.07 ± 0.20), median BMI SDS increased with +0.01 [-0.1 – 0.26] per month. During the first year of treatment (mean year 0.96 ± 0.08), the BMI SDS decreased significantly to -0.05 [-0.29 – 0.04] per month (*p* = 0.006, **Figure 1**). Exemplary BMI trajectories of the treated patients are shown in **Figure 2**.

**Table 2:** Change in BMI SDS over time during dextroamphetamine treatment.

	BMI SDS 12m before start	BMI SDS 6m before start	BMI SDS at start	BMI SDS 6m during treatment	BMI SDS 12m during treatment	BMI SDS at last moment of follow-up
***TOTAL GROUP (n = 17)*** *(mean treatment duration 23.7 ± 12.7 months)*						
Mean BMI SDS ± SD	3.32 ± 1.31	3.54 ± 0.87	3.58 ± 0.85	3.24 ± 1.07	3.01 ± 1.34	3.18 ± 1.44
***RESPONDERS (n = 14)*** *(mean treatment duration 22.4 ± 10.5 months)*						
*Children with BMI decline (n = 11)*						
Mean BMI SDS ± SD	2.89 ± 1.36	3.18 ± 0.63	3.20 ± 0.60	2.68 ± 0.74	2.26 ± 0.90	2.42 ± 1.04
*Children with BMI stabilization (n = 3)*						
Mean BMI SDS ± SD	4.15 ± 0.74	4.32 ± 0.81	4.32 ± 0.94	4.24 ± 0.84	4.29 ± 0.87	4.40 ± 0.95
***NON-RESPONDERS (n = 3)*** *(mean treatment duration 29.7 ± 22.6 months)*						
Mean BMI SDS ± SD	4.10 ± 1.00	4.13 ± 1.09	4.24 ± 0.94	4.25 ± 0.99	4.49 ± 0.76	4.76 ± 1.02

BMI SDS, Body Mass Index standard deviation score; m, months; SD, standard deviation.

When comparing the period of dextroamphetamine treatment to the period without dextroamphetamine using mixed model analysis, a statistically significant decrease in BMI SDS per month was found (ΔBMI SDS –0.03, CI 95% -0.04 to -0.02, *p* < 0.001).

### Effect of Dextroamphetamines on BMI SDS in Subgroups

#### Responders

Of the 17 patients, in total 14 patients (82.4%) were classified as responders. Of these fourteen patients, there were ten patients (71%) with acquired HO, three (21%) with genetic HO, and one (7%) with an anatomical cause of HO.

**Figure 3:**
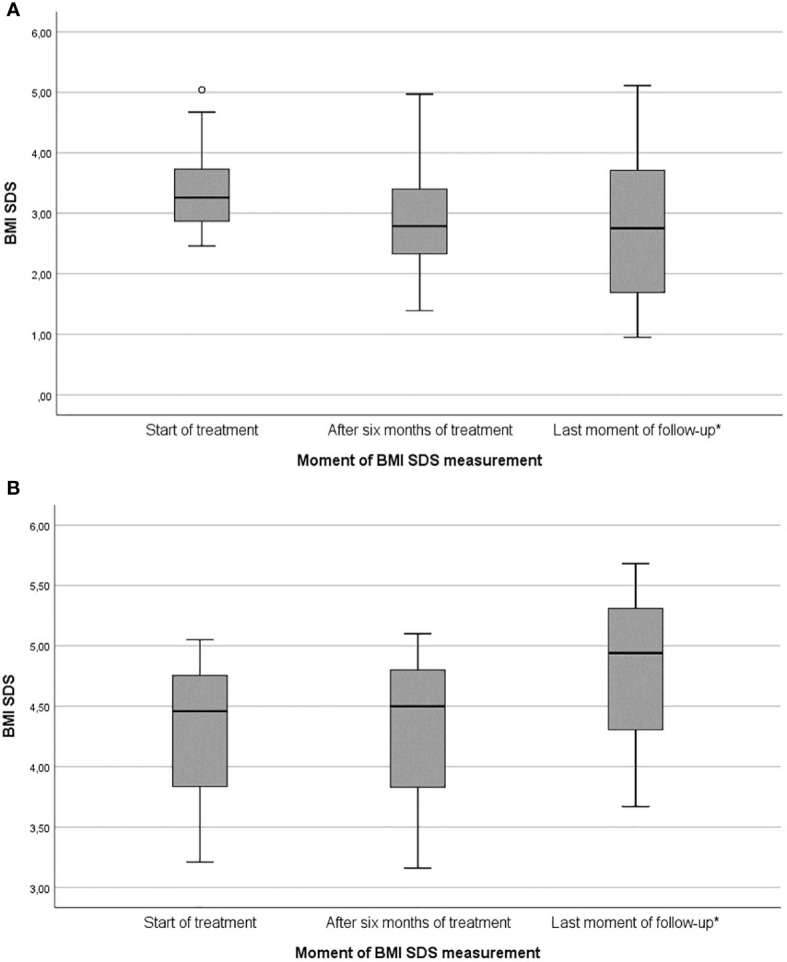
Box plots of changes in BMI SDS during dextroamphetamine treatment for HO: **(A)** Responders subgroup (*n* = 14), **(B)** Non-responders subgroup (*n* = 3). BMI SDS, Body Mass Index standard deviation score. * Mean treatment duration of responders (22.4 ± 10.5 months) and non-responders (29.7 ± 22.6 months).

Of the 14 responders, mean BMI SDS at start of treatment was 3.44 ± 0.80 [2.46 – 5.04], which decreased to 3.02 ± 0.99 [1.39 – 4.97] after 6 months of treatment, and to 2.84 ± 1.30 [0.95 – 5.11] at last moment of follow-up (**Table 2** and **Figure 3**). In the year before start of treatment (mean year 1.08 ± 0.22), median increase in BMI SDS was +0.01 [-0.10 – 0.26] per month. This decreased significantly to -0.05 [-0.29 – 0.01] per month (*p* = 0.004) during the first year of treatment (mean year 0.96 ± 0.08).

Of the 14 responders, in total nine children had been diagnosed with multiple pituitary deficiencies and used hormone replacement. The FT4 concentration was significantly higher during dextroamphetamine treatment in four patients (ID 3. median FT4 17.0 pmol/l [17-22] versus 23.0 pmol/l [18.6 – 24.3], ID 6. FT4 25.0 pmol/l versus 29.0 pmol/l, ID 9. median FT4 14.5 pmol/l[14-15] versus 18.0 pmol/l [17.0 – 21.0], ID 11. Median FT4 19.5 pmol/l [17.9-21.0] versus 23.1 pmol/l [19.60 – 25.5] (*p* = 0.021). IGF-1 SDS concentrations or dosage of hydrocortisone/m^2^ in the 12 months before treatment compared to the period of dextroamphetamine treatment did not differ. One patient started estrogen suppletion during dextroamphetamine treatment.

#### BMI Decline

Of the 14 responders, 11 patients (64.7%) were classified into the BMI decline (BD) subgroup. Their mean ΔBMI SDS after 6 months of treatment was -0.51 ± 0.37, which decreased to -0.78 ± 0.84 at last moment of follow-up (**Table 2**). In the year before start of treatment (mean year 1.03 ± 0.09), median increase in BMI SDS was +0.01 [-0.10 – 0.26] per month. This decreased significantly to -0.05 [-0.29 – -0.03] per month (*p* = 0.006) during the first year of treatment (mean year 0.98 ± 0.07). In seven out of 11 patients (63.6%) who responded with BMI decline, the greatest response was observed in the first six months of treatment (BMI SDS -0.48 ± 0.40 in the first six months vs. BMI SDS +0.17 ± 0.16 during rest of follow-up).

#### BMI Stabilization

Of the 14 responders, three patients (35.3%) were classified into the BMI stabilization subgroup (BS). Their mean ΔBMI SDS after six months of treatment and at last moment of follow up was -0.08 ± 0.16 and +0.08 ± 0.02, respectively (**Table 2**). In the year before start of treatment (mean year 1.25 ± 0.99), mean increase in BMI SDS was +0.01 [-0.01 – 0.03] per month. This decreased to -0.01 [-0.01 – 0.01] per month (*p* = 0.285) during the first year of treatment (mean year 0.91 ± 0.08).

#### Non-Responders

Of the 17 patients, three patients (17.6%, *n* = 2 with acquired HO and *n* = 1 with genetic HO) were classified as non-responders. Mean BMI SDS at start of treatment was 4.24 ± 0.94 [3.21 – 5.05], which increased to 4.25 ± 1.00 [3.16 – 5.10] after six months of treatment and to 4.76 ± 1.02 [3.67 – 5.68] at last moment of follow-up (**Table 2** and **Figure 3**). In the year before start of treatment (mean year 1.01 ± 0.07), median increase in BMI SDS was +0.01 [0.00 – 0.02] per month. This increased to +0.02 [0.01 – 0.04] per month (*p* = 0.285) during the first year of treatment (mean year 0.93 ± 0.10).

### Effects on REE and Body Composition

Of the 17 patients, REE was measured before and during treatment in 13 children. Mean measured REE divided by predicted REE (mREE/pREE) before treatment was 69.7% ± 20.2 and mean mREE/pREE during treatment was 80.6% ± 15.7 (after mean months 11.2 ± 8.3). In 11/14 responders (BD *n* = 8, BS *n* = 3), a mREE/pREE increase of +8.9% ± 14.2 was seen (**Table 3**). In 5/8 (62.5%) patients of the BD subgroup (ΔBMI SDS ≥ -0.25), REE increased with ≥10% during treatment. In 3/3 patients of the BS subgroup (-0.1 to 0.1 ΔBMI SDS), REE did not change during treatment (decrease mREE/pREE of -0.43% ± 5.3). Of the two non-responders, increase of MREE/pREE +29.0% and +14.6% after 30 months and six months of treatment, respectively (**Table 3**).

**Table 3:** Resting energy expenditure and body composition at start and during dextroamphetamine treatment.

Patient ID	*m*	REE^ (% of predicted) before start^a^	*m*	REE (% of predicted) at follow-up	*m*	Fat mass^^ (%) before start	*m*	Fat mass (%) at follow-up
***RESPONDERS***								
*Children with BMI decline*								
2	-2	63.5	+7	79.5	-2	28.8	+7	32.9
3	-1	42.8	+22	75.8	-1	32.5	+22	27.2
4		n.a.	+56	83.4		n.a.	+54	47.9
5	-7	52.0	+7	79.0	-7	20.4	+7	24.8
6		n.a.	+37	90.0		n.a.	+37	30.6
7	-2	43.8^b^	+9	40.6		n.a.	+10	38.8
8	0	51.3	+11	64.0	0	51.9		n.a.
9	-12	63.0	+12	82.9	-12	34.4	+12	31.1
11	-4	90.3	+3	96.4	-2	53.3	+3	49.3
12		n.a.	+5	56.9		n.a.		n.a.
17	-3	106.9	+14	94.7	-3	55.0	+14	51
*Children with BMI stabilization*								
15	-1	96.4	+18	90.4	-1	58.0	+1^c^	60.3
16	-5	67.2	+3	71.8		n.a.		n.a.
19	-2	84.3	+3	84.4	-2	54.9	+3	56.0
***NON RESPONDERS***								
1	-50	69.4	+30	98.4	-3	51.1	+30	50.6
10	0	75.4	+6	90.0	0	48.5	+6	48.5
13	-9	93.0		n.a.	-9	44.0	+33	43.5

REE, resting energy expenditure; n.a., not available; m, months, minus sign is before treatment, plus sign after start of treatment.

ID 14 and ID 18 were excluded due to short treatment duration or the simultaneous start of extensive lifestyle treatment.

^ Resting energy expenditure was measured after an 8-h overnight fast and after a 30 minute resting period, using indirect calorimetry. This was done with either the Cosmed Quark RM (Cosmed, Italy) or Geratherm Ergostik (Geratherm Respiratory GmbH, Germany).

^^ FM was measured using dural energy X-ray absorptiometry scans, air displacement plethysmography (BOD POD, Cosmed, Italy) or body impedance analysis (using the Bodystat 1500 or Tanita).

^a^Predicted REE was calculated using the Schofield formula; ^b^Possible overestimation due to agitation of patient; ^c^Measured one month after stop of treatment.

Of 17 patients, Fat Mass percentage (FM%) was measured in 11 (64.7%) children before and during treatment. Mean FM% before treatment was 43.7% ± 12.7 and mean FM% during treatment was 43.2% ± 12.2 (after mean months 14.1 ± 10.7). In 8/14 of the responders (BD *n* = 6, BS *n* = 2), a mean FM% difference of +0.6% ± 4.0 [-4.4 – 5.3] was seen (**Table 3**). In the non-responders (*n* = 3), a mean FM% difference of +0.1% ± 1.7 was seen (**Table 3**).

### Effects on Behavior and Hyperphagia

Of all 17 treated patients, ten (58.8%) reported improvement regarding satiety and/or hyperphagia. Patient reported behavior, e.g. food stealing or seeking behavior, concentration level, or number of rage attacks, improved in eight patients (47.1%) and patient reported activity level improved in five patients (29.4%). One patient continued treatment, even though no effects on weight were observed, because of subjectively reported improved duration and quality of sleep. Of the eleven responders with a BMI decline, 45.5% reported a reduction of the effect on the earlier improved hyperphagia or behavior over time. This reduction of effect of dextroamphetamine over time was present in both children with acquired HO as well as genetic HO.

### Adverse Effects

Of the 19 patients treated with dextroamphetamines for HO, two patients developed hypertension during dextroamphetamine treatment (systolic blood pressure >95^th^ percentile). For one patient with genetic HO, this led to early termination of treatment after one month due to the stage 1 hypertension, together with the lack of effect on hyperphagia and weight. The other patient, also with genetic HO, developed stage 2 hypertension at a daily dose of 15 mg per day, which resolved when the dose was reduced to 10 mg per day on which the patient continued treatment. It was, however, questionable whether an appropriately sized cuff was used during the first measurements. For both patients, there was no need to start antihypertensive treatment. Four patients (23.5%) reported newly experienced difficulties falling asleep. Two patients reported negative changes in behavior. In one patient dextroamphetamine resulted in a flattened effect of emotions and hypo-activity and in the other hyperactivity and anger, which improved after reducing the dose. This resulted for both patients in discontinuing of treatment after nine months and two and a half years, respectively.

## Discussion

We have described our experiences with dextroamphetamines in a retrospective cohort, which is the largest cohort studied until now, and found positive effects of dextroamphetamine treatment on BMI in 82.4% of children with HO. In addition, 76.5% of the children reported an improvement of hyperphagia, behavior, and/or energy level, which was of major importance for most of the families. At the end of the follow-up period, 70.6% of children wanted to continue treatment. These results, which need confirmation in larger cohorts, suggest that dextroamphetamine treatment may be a beneficial addition to lifestyle interventions for children and young adolescents with HO.

Our results are in line with the reports of three previous smaller cohorts (*n* = 5, *n* = 7, and *n* = 12) of children with acquired HO (26–28). When pooling the results of these previous studies, 22 of the 24 included patients (92.0%) demonstrated either weight reduction or stabilization (26–28). The first study of Mason et al. reported stabilization of BMI in five children during dextroamphetamine treatment. Moreover, all children reported improved overall activity and behavior (26). The second study of Ismail et al. reported weight reduction or stabilization in 10 out of 12 children and in 11 out of 12 children an improvement of subjectively reported daytime somnolence possibly leading to increased mobility (27). The third and most recent study of Denzer et al. reported reduction or stabilization of BMI SDS in all seven patients with HO (28). All three studies included patients with similar underlying causes of acquired HO as our patients with acquired HO included in this study. Considering the devastating natural course of HO which includes a progressively increasing BMI, the positive results of all four studies, including ours, are promising. To our knowledge, studies on the effects of dextroamphetamine treatment in patients with genetic HO are lacking. However, there have been two studies in which 6 children with monogenic obesity were successfully treated with methylphenidate, a psychostimulant with similar characteristics and side effects, such as disordered sleep or negative behavioral changes (34, 35). Our study suggests that dextroamphetamine treatment may be beneficial for patients with genetic HO as well.

This is the first study describing the effects of dextroamphetamines on REE in children with HO, reporting a mean mREE/pREE increase of 8.9% ± 14.2 in the responder (BMI decline and stabilization) subgroup. Moreover, in 62.5% of the patients who responded with BMI decline, an increase of REE of more than 10% was observed. This increase might be explained by the fact that amphetamines are sympathomimetic drugs which increase energy expenditure *via* different mechanisms, such as an increased adrenergic tone, increased serotonin availability and direct stimulation of CART neurons of the leptin-melanocortin pathway (25, 36, 37). The effects of dextroamphetamines on the leptin-melanocortin pathway could perhaps explain why some patients with genetic HO in our cohort did not respond with BMI decline or did not show an effect on REE during dextroamphetamine treatment, as most of these patients have genetic disruptions in this specific pathway. It was remarkable that two non-responders with acquired HO also had increased mREE/pREE which did not positively seem to affect BMI. It must be mentioned that these two patients had the most severe HD, together with high caloric feeding and an inactive lifestyle during treatment. In addition, the period between the REE measurements before and during treatment for one patient was relatively long (i.e. 80 months) and measured with two different devices, so interpretation should be made with caution. Further studies are needed to investigate the relationship between REE and dextroamphetamine treatment in HO.

One of the strengths of our study is our sample size, which is, although small, the largest that has been reported until now. Moreover, our study included patients with acquired HO, as well as congenital HO. Additionally, we evaluated the effect of dextroamphetamines on REE and body composition which have not earlier been reported. Because of the rareness of HO, international multicenter studies should be initiated to increase sample size and to set up a prospectively designed phase II study with objectively measured outcome variables (e.g. standardized questionnaires for behavior) to verify our results.

Several limitations of our study should be considered, such as the retrospective study design, the presence of other interventions in some of our patients, the lack of a control group, and subjective outcome measures. However, large placebo-controlled randomized clinical trials may not be feasible due to the rareness of HO, which highlights the importance of observational studies. The differences in dextroamphetamine dosages and treatment duration complicated the interpretation of BMI SDS changes in our study. To overcome this difference in treatment duration, a mixed model analysis was done taking longitudinal data into account. Because of the complexity and severity of HO, some of the patients were treated with other pharmacotherapies, such as glucagon-like-peptide 1 agonists or oxytocin, or specialized dietary treatment simultaneously. These simultaneous treatments may have influenced their BMI. We excluded one patient for BMI analyses because of a simultaneous start of extensive lifestyle intervention (for the first time) at a rehabilitation clinic and dextroamphetamine treatment. All other patients with concurrent pharmacotherapies or dietary intervention did not simultaneously start with this treatment and could therefore be included in the analysis. This is illustrative of the complexity of children with HO. In addition, most children with acquired HO received pituitary replacement therapy. Four responders showed a significant increase in FT4 concentration during treatment with dextroamphetamine, which may have been confounding. However, these FT4 measurements need to be interpreted with serious caution, as they were not systematically determined and, more importantly, several different laboratory assays were used, making the values difficult to compare.

The timing of dextroamphetamine treatment in our cohort of children was not formally defined upfront, so this might hamper optimal results. Some children in this cohort, especially the patients with congenital HO and some with acquired HO, had already been diagnosed with HO for several years. Perhaps this prolonged follow-up phase before start of dextroamphetamine treatment may have negatively affected the effects of dextroamphetamine treatment. Future studies are needed to address the question whether treatment with amphetamines earlier in follow-up or even at diagnosis of HO might be more beneficial to prevent or decrease weight gain. Another less desirable effect of amphetamines is drug tolerance or habituation, in which over time less effect is observed. The three children who did not respond on dextroamphetamine therapy, did not show signs of drug tolerance. However, in seven of 11 patients who responded with BMI decline, the greatest response was observed in the first six months of treatment. Additionally, six of these seven patients reported deterioration of the earlier improved hyperphagia and behavior over time. This opens the debate upon the optimal dosing regimen and the consideration to introduce intermittent therapy with short periods of “wash out” in these patients.

Pharmacotherapy, such as with dextroamphetamines, may be considered as an off-label treatment option, in addition to lifestyle interventions (e.g. personalized dietary and/or physical activity interventions, such as low carbohydrate diet, high-intensity training, or intensive coaching trajectory in a rehabilitation clinic) in patients with HO. This can be prescribed for a testing period, preferably for at least three to six months, after which its effect can be evaluated. Such a testing period could be helpful to identify the patients who would benefit of such treatment (e.g., reduction or stabilization of weight, improvement of hyperphagia or behavior). We advocate, while awaiting of placebo-controlled studies, to consider a role of dextroamphetamine, next to lifestyle interventions, in individual cases. REE measurements before and at six months after dextroamphetamine treatment are advised. The likelihood of a positive response to dextroamphetamine treatment may be increased when low REE together with hyperphagia is present. In future studies, combinations of drug treatments, such as dextroamphetamine together with GLP-1 analogues, may be investigated. GLP-1 analogues, in combination with dextroamphetamine, may interfere or activate different neural secretory pathways, and therefore possibly a synergistic effect might be observed. In addition to the effects of dextroamphetamine, GLP-1 analogues slows gastric emptying and increase satiety, leading to a lower food intake (38). GLP-1 analogues have recently shown to be effective in reducing weight and hyperphagia in four adult patients with genetic HO (39).

The pathophysiology of genetic and acquired HO is different from other forms of refractory obesity. Studies have shown good effects of amphetamines in treating obesity, however it also comes with serious side effects (40). The main concerns with amphetamine relate to toxicity, addictive potential, and concern that the small increase in blood pressure could exacerbate ischemic heart disease. In the 1950s, amphetamines were often used for patients with common obesity, leading to psychosis and malnutrition, together with depression on drug withdrawal. Subsequently, prescribing of amphetamines became restricted (20). In this study, all children were treated using the dosage regimen which is also used in the treatment of ADHD. Long-term studies evaluating the safety profile and side effects of dextroamphetamine treatment in children with ADHD have not shown considerable safety issues (41). Therefore, we believe that dextroamphetamines may be indicated and helpful in patients with specific obesity disorders, who suffer from uncontrollable weight gain due to hyperphagia and/or low resting energy expenditure. This can be an obesity disorder such as HO, but perhaps also other forms of refractory obesity with the same phenotype. However, it should be noted that dextroamphetamine treatment should only be considered in individual cases and in a multidisciplinary expertise setting and after lifestyle interventions have been started.

In conclusion, our study supports the potential positive role of dextroamphetamine treatment for hypothalamic obesity in children and young adolescents. Dextroamphetamine treatment may lead to BMI decline or stabilization and improvement of hyperphagia and/or behavioral problems during long-term follow-up, as is shown in our cohort. For all children with obesity, including hypothalamic obesity, lifestyle intervention remains the cornerstone of treatment. The addition of dextroamphetamines for this indication should only be considered in individual cases and in a multidisciplinary setting with expertise on HO and after lifestyle interventions have been started. Prospective treatment trials are needed to further elucidate the efficacy of dextroamphetamine treatment on weight.

## Data Availability Statement

Restrictions apply to the availability of some or all data generated or analyzed during this study to preserve patient confidentiality or because they were used under license. The corresponding author will on request detail the restrictions and any conditions under which access to some or all data may be provided.

## Ethics Statement

The studies involving human participants were reviewed and approved by Medisch Ethische Toetsingscommissie Utrecht and Medisch Ethtische Toetsingscommissie Erasmus Medisch Centrum Rotterdam. Written informed consent was obtained from the individual(s), and minor(s)’ legal guardian/next of kin, for the publication of any potentially identifiable images or data included in this article.

## Author Contributions

JvS and MW made equal substantial contributions to the conception and design of the work, the acquisition, analysis, interpretation of the data, and drafted the work. These authors share first authorship. CdG, JvE, AJ, MB, BO, BB, WT, and NS made substantial contributions to the analysis and interpretation of the data, and critically revised the work. EA and HvS made equal substantial contributions to the conception and design of the work, the acquisition, analysis, interpretation of the data, and critically revised the work. These authors share last authorship. All gave final approval of the version to be published and agreed to be accountable for all aspects of the work in ensuring that questions related to the accuracy or integrity of any part of the work are appropriately investigated and resolved.

## Conflict of Interest

The authors declare that the research was conducted in the absence of any commercial or financial relationships that could be construed as a potential conflict of interest.

## Publisher’s Note

All claims expressed in this article are solely those of the authors and do not necessarily represent those of their affiliated organizations, or those of the publisher, the editors and the reviewers. Any product that may be evaluated in this article, or claim that may be made by its manufacturer, is not guaranteed or endorsed by the publisher.
